# Supplementary material for: Assessing the appropriateness of the Moving Epidemic Method and WHO Average Curve Method for the syndromic surveillance of acute respiratory infection in Mauritius
Source: PLoS One. 2021 Jun 3;16(6):e0252703. doi: 10.1371/journal.pone.0252703 (PMC8174728; doi:10.1371/journal.pone.0252703)
Supplement: Attachment — Submitted filename: Response to Reviewers.docx [file pone.0252703.s003.docx]

Fernanda C. Dórea

Academic Editor

PLOS ONE

24^th^ April 2021

Dear Dr. Dórea

Thank you for considering our manuscript for publication and very insightful reviews that we have addressed as shown below. We understand that it has been very hard to find reviewers and we would like to express our gratitude for taking the time to appraise our manuscript.

Thank you for your consideration.

Kind regards

Mohabeer Teeluck

Epidemiologist/Senior Epidemiologist

Ministry of Health and Wellness, Mauritius

**Response to Reviewers**

**Methods**

**Question:** Line 117 As the data are percentages it is correct the use of the point interval of the arithmetic mean for MEM Intensity thresholds. But you should explain why the Epidemic/seasonal threshold is calculated through the median and Nyblon CI when the arithmetic mean is used (and recommended) in references 14 & 15. It is important also to mention the slope parameter used in the MEM model, and if this parameter was optimized of it has been selected manually.

**Answer:** Thank you for your review. In reference 14, Vega et al stated the following in the Discussion that “In this work, we chose the parameters which, based on the authors experience, would give the most reliable results. **However, these parameters may be adjusted by countries: more/less pre‐epidemic points, median and bootstrap confidence intervals, arithmetic or geometric means, etc. The variations in these parameters do not question the underlying structure of the model and allow the method to be adapted by different surveillance systems.**”

We chose the median and Nyblom CI as we felt it would be more appropriate for comparison of the MEM with the WHO Average Curve method (which calculated the epidemic thresholds using the median formula on Excel spreadsheet) and the fact that we had flexibility to choose this parameter based on the fact that the sample size was greater than 5 (This link https://rdrr.io/cran/mem/man/memmodel.html, the following was stated in the section details at line 13 “Option 3 uses the Hettmansperger and Sheather (1986) and Nyblom (1992) method, when there is enough sample size.” ).

I would also emphasized that as stated above, that the choice of the parameters can be adapted to our surveillance system and there was no specific recommendation based on the manual for MEM (The MEM Web Application: technical manual Reference 21).

**Question:** Line 154. The title of the subsection ‘Sensitivity analysis without the year 2017’ can be confuse. Actually, it is the comparison of the model indicators with and without the year 2017. Maybe ‘Comparison analysis…….’ Anyway, in my opinion PropARI in 2017 are no exceptional: peak around 0.3 (0,2 in 2014), and the authors could consider to present only the model with all the seasons. Or, at least, discuss this point in the discussion section. In this way, the intensity thresholds will reflect better the ARI pattern in the country (the epidemic thresholds and the cross-validation indicators would not change, as the analysis confirm).

**Answer:** Thank you for your review. We can understand the editor’s concern that the wording of ‘sensitivity analysis’ is confusing. Thus, we have agreed to replace ‘sensitivity analysis’ with ‘comparative analysis’ here as well as in other parts of the manuscript.

With regards to why we decided that that a peak of 0.3 was exceptional, we even analysed ARI data going back to 2007, and the only time we did find a peak above 0.3 was 0.36 during week 34 in 2009 (N.B: we were in the middle of the influenza H1N1 pandemic of 2009). Therefore, we decided that based on the fact that this event prompted public health actions to be taken by authorities in the country, we compare both models with 2017 and without as a precautionary measure. Therefore, we would appreciate if we could go ahead with the content as it is but as the editor proposed to replace the sensitivity analysis with comparative analysis.

**Question:** Line 176. Statistical analysis. Do you assume that ‘normality’ exists in the cross validation indicators to use the t-test? Please discuss it.

**Answer:** Thank you for your insightful review, and based on the fact that the indicators did not follow normality, we have decided to use a non-parametric testing and therefore for this study we chose the Wilcoxon-rank test for all indicators.

**Results**

**Question:** Line 201. Maybe a mistake: The epidemic period for the 2WS curve for MEM (23 weeks) was a week shorter than the associating curve (22 weeks) of WHO ACM.

**Answer:** Thank you. Corrected. It should have been “The epidemic period for the 2WS curve for MEM (23 weeks) was a week shorter than the associating curve (24 weeks) of WHO ACM.

**Question:** I would suggest to combine table 2 and table 3 to better describe the text from line 264.

Answer: Table 2 and Table 3 have been merged as recommended.

**Question:** Line 325. It is not clear for me this sentence, since the difference of seasonal thresholds is 0.: Therefore, it is nearly inevitable to observe overlapped confidence intervals of the 2WS curves for WHO ACM and WHO ACM w/o 2017, regardless of the p-value.

Could you explain it a little more?

**Answer:** We agree with the assessment of the reviewer that the sentence was not clear and confusing. Therefore we changed the sentence to the following ‘‘Therefore, it was statistically not feasible to verify any significant differences between the 2WS curves for WHO ACM and WHO ACM w/o 2017.’’

**Additional Editor Comments:**

Line 20: remove "system" (or it should be "a system", with "a" added at the beginning of the sentence)

**Corrected as requested**

Line 25: yearly?

**I have removed the word “weekly” and it’s not yearly. Basically, the proportion of attendance for acute respiratory infections was selected as the transmissibility indicator in this study, to describe seasonality.**

Line 26: proportion of attendance of what? Some more is explained later in the same paragraph, so maybe just review the paragraph to clarity. Also, attendance "due to" or "attributed to", not "for acute"

**Corrected as requested.**

Line 34: W/S not explained before what it is.

**Corrected as requested.**

Methods:
Virological data: you don't need a session for something that was not included. You can just mention at the end of the previous paragraph that this was not considered viable to include.

**Corrected as requested.**

**Question:** The figures attached had very poor quality, hindering review. The authors mention a lot the 2 waves characteristic of the temporal trend for ARI, I think this should be clearly shown with a plot that has data for all years in the same plot. Maybe the raw data can be presented as a time-series in Figure 1. Preferentially as a bar chart of all consultations (denominator), with a line superimposed showing the proportion attributed to ARI.

**Answer: New figures uploaded as time-series for figure 3 and figure 4.**

Please review the writing. The English language, but also the general writing clarity needs to be improved.

Abstract and Introduction need rewriting to improve style and clarity.

**Answer:** We went through the manuscript again and made necessary corrections and did a thorough a grammar check.
